# Investigation of Corrosion Resistance of 60Si2MnA Spring Steel Coated with Zn-Al in Atmospheric Environments

**DOI:** 10.3390/ma18143215

**Published:** 2025-07-08

**Authors:** Yurong Wang, Hui Xiao, Baolong Liu, Shilong Chen, Xiaofei Jiao, Shuwei Song, Wenyue Zhang, Ying Jin

**Affiliations:** 1 National Center for Materials Service Safety, University of Science and Technology Beijing, Beijing 100083, China; wangyurong@ustb.edu.cn (Y.W.);; 2 Beijing Institute of Space Launch Technology, Beijing 100076, China

**Keywords:** 60Si2MnA spring steel, Zn-Al coating, indoor accelerated corrosion, atmospheric corrosion, corrosion resistance

## Abstract

To investigate the corrosion resistance of 60Si2MnA spring steel coated with Zn-Al in a domestic atmospheric environment containing harmful salts, the corrosion environmental factors (temperature, humidity, deposited salts, and pH) were obtained through field research. The deliquescence and weathering behavior of harmful salts were studied using impedance methods to establish their characteristic curves. Additionally, a self-designed salt deposition test apparatus was employed to conduct accelerated atmospheric corrosion tests under constant salt deposition (10 g/m^2^) and controlled temperature and humidity conditions (20 °C/75% RH and 40 °C/75% RH) over different corrosion periods. The results show that noticeable red rust appeared on the samples after one month of corrosion. As the temperature increased, the consumption of the coating accelerated. XRD and Raman analyses reveal that the main corrosion products of the coating materials were ZnO, Zn(OH)_2_, and Zn_5_(CO_3_)_2_(OH)_6_, while the red rust primarily consisted of iron oxides and hydroxides. In the early stages of corrosion, the self-corrosion current density was relatively low due to the protective effects of the coating and the corrosion product layer, indicating good corrosion resistance. However, in the later stages, the integrity of the coating and the corrosion product layer deteriorated, leading to a significant increase in the self-corrosion current density and a decline in corrosion resistance. This study provides a data foundation for understanding the corrosion behavior of Zn-Al-coated spring steel in atmospheric environments and offers theoretical insights for developing more corrosion-resistant coatings and optimizing anti-corrosion measures.

## 1. Introduction

60Si2MnA spring steel is widely used in automobile manufacturing and aerospace and engineering machinery as a metallic material with excellent mechanical properties, creep resistance, and fatigue resistance [1]. Its chemical composition contains silicon, manganese, and a small amount of carbon, where the content of Si improves the elasticity and wear resistance of the steel but reduces the substrate electrode potential, while the presence of Mn effectively enhances its tensile strength and fracture toughness but is prone to lead to the formation of microcells in the formation of segregation, accelerating electrochemical corrosion. In particular, in humid conditions, salt spray, or acidic media, the surface is prone to oxidation and corrosion [2,3], threatening the life of components and their safety.

To address the corrosion issues of metallic materials in service environments, various surface treatment technologies, such as organic and metallic coatings, have been widely applied to different metal structures to maintain high corrosion resistance over long-term use [4,5,6]. Coatings primarily function by isolating the metal from direct contact with corrosive environments, providing a physical barrier. Some coatings can also enhance corrosion resistance through chemical interactions. Currently, metal surface protection technologies mainly include spraying, hot-dip coating, and painting processes. These surface treatment techniques have significantly improved the corrosion resistance of metallic materials, particularly in harsh environments, where coatings effectively slow down the corrosion process [7,8], thereby extending the service life of the material. However, coating protection does not completely prevent corrosion in all cases. In specific atmospheric environments, especially those with high humidity, high salt spray, and severe industrial pollution, thin liquid films form on metal surfaces, leading to electrochemical reactions [9]. Even with anti-corrosion coatings, issues such as peeling and cracking may occur, exposing the metal surface and initiating corrosion. Zou et al. [10]. studied the corrosion behavior and electrochemical performance of arc-sprayed Al, Zn-Al alloy, and Zn-Al pseudo-alloy coatings in a 3.5 wt.% NaCl solution, finding that pitting corrosion on the aluminum coating surface led to substrate corrosion. Katayama et al. [11]. investigated the corrosion behavior of carbon steel with thermally sprayed Zn, Al, and Zn-Al coatings exposed to coastal environments for 33 years using electrochemical impedance tests. They demonstrated that the long-term corrosion resistance of Zn and Al coatings increased with initial coating thickness, and the selective dissolution of Zn promoted the formation of thicker corrosion products, enhancing corrosion resistance. Li et al. [12]. prepared Zn-xAl composite coatings with varying aluminum content and studied their corrosion behavior in a 3.5 wt.% NaCl solution through electrochemical tests. They found that the corrosion resistance of the composite coatings significantly improved with increasing aluminum content.

Existing research predominantly employed single-factor accelerated test methods, such as salt spray or solution immersion [13], which failed to fully reveal the corrosion behavior characteristics under the coupled effects of multiple factors in real atmospheric environments. Therefore, this study integrated atmospheric corrosion conditions and focused on a specific region in China with a harmful salt-containing atmosphere. Using spring steel coated with Zn-Al as the research subject, the field environmental parameters (temperature, humidity, pH value, and salt deposition) were obtained through environmental investigation. Accelerated corrosion tests under constant temperature and humidity conditions over different corrosion periods were conducted [14]. Techniques such as weight loss analysis, surface macro- and micro-morphology observation, corrosion product identification, and electrochemical testing were employed to analyze the variations in corrosion behavior at different temperatures (20 °C and 40 °C) and corrosion periods (1, 2, 3, and 6 months). This study aimed to reveal the corrosion failure mechanisms of Zn-Al-coated spring steel in harmful salt-containing atmospheric environments.

By conducting in-depth research on the corrosion mechanisms of 60Si2MnA spring steel coated with Zn-Al in various atmospheric environments, theoretical foundations can be established for optimizing anti-corrosion measures. This will aid in designing more efficient and durable anti-corrosion coatings, thereby enhancing the service life of spring steel and reducing economic losses caused by corrosion.

## 2. Experiment

### 2.1. Materials and Sample Preparation

The material used in the experiment was 60Si2MnA spring steel coated with Zn-Al, with the following chemical composition (wt.%): C 0.58, Si 1.66, Mn 0.86, S 0.0045, P 0.0186, Cr 0.1340, Ni 0.1040, and Cu 0.0058. The specimens were processed into 100 mm × 50 mm × 2 mm flat plates. After weighing, the specimens were placed in a drying oven (50 °C/10% RH) for storage.

The surface Zn-Al coating treatment process for the arc spraying includes the following: an arc zone temperature > 5000 °C, a spraying distance of 150–200 mm, a carrier gas type for compressed air (dew point < −40 °C), a powder particle size of 45–75 μm, a powder feeding rate of 20–30 g/min, and the main components for the Zn powder and a small amount of Al powder. When the material is in the environment of the corrosive medium, the metal in the coating (mainly Zn) plays the cathodic protection role of a sacrificial anode to the substrate.

### 2.2. Indoor Accelerated Corrosion Test

In response to the atmospheric corrosion environment containing harmful salts in a certain region of our country, environmental parameters were obtained through on-site research: the temperature range is 20~40 °C, the humidity is 75% RH, and the salt deposition amount was measured to be 1~10 g/m^2^ using an Elcometer 138 Bresle Salt Kit with salt patches on-site. At the same time, the precise pH value was measured to be 4.5 using pH test strips. The experiment selects extreme temperature values of 20/40 °C, an extreme salt deposition amount of 10 g/m^2^, and a pH of 4.5. Based on the deliquescent weathering laws of mixed salts and service time, indoor corrosion experiments were designed to simulate actual corrosion environment conditions in order to replicate field environmental characteristics and study material degradation behavior. The experimental solution primarily contained cations, such as Ca^2+^, Na^+^, K^+^, and Mg^2+^, and anions, such as Cl^−^, HCO_3_^−^, SO_4_^2−^, and NO_3_^−^. Among these, Ca^2+^, Na^+^, Cl^−^, and SO_4_^2−^ were the most abundant ions, with corrosiveness mainly associated with Cl^−^ and SO_4_^2−^. The salt deposition solution used in the tests had a pH value of 4.5, and the amount of salt deposited on the specimen surfaces was fixed at 10 g/m^2^. This study focused on the corrosion behavior of specimens deposited with harmful salts under conditions of 20/40 °C and 75% relative humidity (RH) for periods of 1, 2, 3, and 6 months.

In order to control the pH of the solution after deliquescence and avoid the introduction of non-systemic ions as much as possible, the acidic conditions were regulated by adding a NaHSO_4_ solution. A salt-settling device was designed and constructed independently to make the mixed salt settle on the surface of the specimen in the form of solid powder to control the test conditions more accurately.

### 2.3. Weight Loss Analysis

For each temperature and humidity condition, four specimens were prepared. Among these, three specimens were used as parallel samples for weight gain analysis, and one specimen was utilized to characterize the post-corrosion morphology, corrosion product composition, and electrochemical testing. After corrosion, the specimens were rinsed with deionized water, and any remaining mixed salt deposits on the surface of the flat samples were gently removed using a soft brush. They were then rinsed a second time with anhydrous ethanol, dried with air, and transferred to a drying oven (50 °C/10% RH) for 24 h before being weighed. The dried samples were weighed on a 0.1 mg precision electronic balance. The weight gain data for each group was calculated as the arithmetic average of the three parallel specimens.

### 2.4. Characterization

The corrosion behavior of the samples under different conditions was systematically characterized using a combination of advanced techniques. First, a digital camera was employed to capture the macroscopic morphology of the corroded samples. The microstructural features and elemental distribution of the corroded specimens were analyzed using a field emission scanning electron microscope (SEM, Merlin Compact, German Zeiss) equipped with an energy-dispersive X-ray spectroscopy (EDS, Oxford) system. X-ray diffraction (XRD) was utilized to determine the phase composition of the corrosion products by analyzing the diffraction patterns. The sample was 1 cm × 1 cm, and Bruker-Bruker D8 Advance (Germany) was used with the following test parameters: an operating voltage of 40 kV, an operating current of 40 mA, a detector-to-sample distance of 280 mm, a scanning angle of 20–90°, a step size of 0.02°, and a scanning speed of 5°/min. Additionally, confocal Raman spectroscopy was applied to obtain the molecular vibration characteristics of the corrosion products through Raman shifts, enabling the identification of their chemical composition and crystal structure. The sample was 1 cm × 1 cm, and the device model was WTTech Alpha 300RA.

### 2.5. Electrochemical Tests

In order to evaluate the protective performance of the corrosion products after the corrosion of 60Si2MnA with a Zn-Al coating, electrochemical testing was conducted on samples with rust layers subjected to corrosion at different temperatures and periods. Based on the findings from the investigation and analysis of atmospheric environments containing harmful salts, the main cations used in the solution were Ca^2+^, Na^+^, K^+^, and Mg^2+^, while the anions were Cl^−^, HCO_3_^−^, SO_4_^2−^, and NO_3_^−^. Considering the minor contribution of precipitates to the corrosion process under static conditions and the influence of corrosion test duration on experimental data, special emphasis was placed on extracting the primary salt components and their ratios. A simulated solution of 0.4 mol/L NaCl + 0.2 mol/L Na_2_SO_4_ was prepared, and electrochemical testing was conducted under ambient temperature conditions to more accurately reflect the actual corrosion mechanisms. The experiment utilized a Gamry Reference 600 electrochemical workstation combined with a standard three-electrode system to analyze the protective performance of corrosion products on metals. After stabilizing the open circuit potential (OCP) of the sample surface for 60 min, a ±5 mV sinusoidal potential perturbation was applied within a frequency range of 10^5^ Hz to 0.01 Hz for electrochemical impedance spectroscopy (EIS) testing, with a scan rate of 0.5 mV/s and a scanning interval from −0.3 V to 0.8 V relative to the OCP.

## 3. Results and Discussion

### 3.1. Characterization of the Original Sample

Experimental samples were prepared in accordance with ISO 4287:1997 the standard requirements of the unified preparation [15]. Each set of experiments consisted of three parallel samples to ensure the reliability of the data.

**Figure 1:**
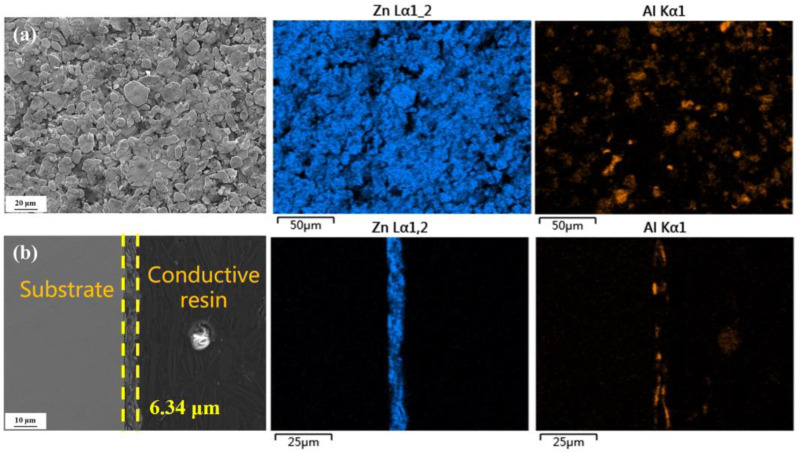
Surface and cross-sectional morphologies of Zn-Al-coated samples, along with EDS spectrum distribution. (**a**) Surface morphology; (**b**) Cross-sectional morphology.

The surface and cross-sectional microstructures of the Zn-Al-coated spring steel were characterized using scanning electron microscopy (SEM), as shown in Figure 1. The sample surface is densely covered with lamellar structures ranging in size from several micrometers to tens of micrometers, exhibiting a typical overlapping distribution pattern. No significant defects, such as cracks or pores, were observed on the surface, indicating excellent structural integrity. The interface between the coating and the metal substrate is tightly bonded, presenting a continuous and uniform morphological characteristic. The average thickness of the coating is approximately 6.343 μm.

### 3.2. Corrosion Weight Change

**Figure 2:**
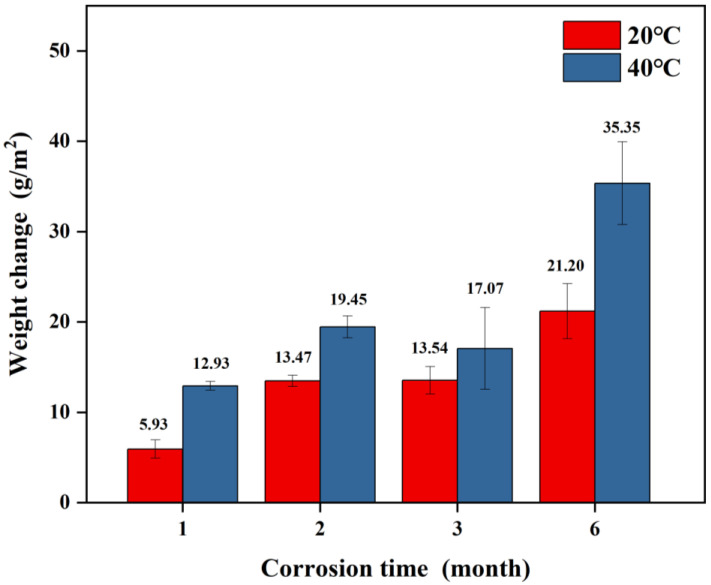
Variation in corrosion weight of Zn-Al-coated samples over time.

Figure 2 illustrates the variation in weight of spring steel samples coated with Zn-Al as a function of corrosion time under two temperature conditions: 20 °C/75% RH and 40 °C/75% RH. The experimental results demonstrate that under constant temperature conditions, the weight gain of the samples significantly increases with prolonged corrosion time. In the 20 °C/75% RH corrosion environment, the weight gain of the samples progressively rose from 5.93 g/m^2^ after one month to 21.20 g/m^2^ after six months. Conversely, under the 40 °C/75% RH condition, the weight gain for the same corrosion duration (six months) reached 35.35 g/m^2^, marking a 66.7% increase compared to the 20 °C environment. The weight change of the samples after three months does not show a significant difference compared to that after two months. It is hypothesized that the reason for this may be the high humidity of the air during salt deposition and the unevenness of the mixed salt powder during settling, resulting in low salt deposition on the surface of the specimen.

Comparing the weight gain of samples under the same corrosion duration, the weight gain at 40 °C consistently exceeded that at 20 °C. Specifically, after six months of corrosion, the weight gain in the 40 °C environment is approximately 66.7% higher than that in the 20 °C environment. This phenomenon indicates that an increase in temperature significantly accelerates the corrosion kinetics process [16].

### 3.3. Macroscopic Morphology of Corrosion

**Figure 3:**
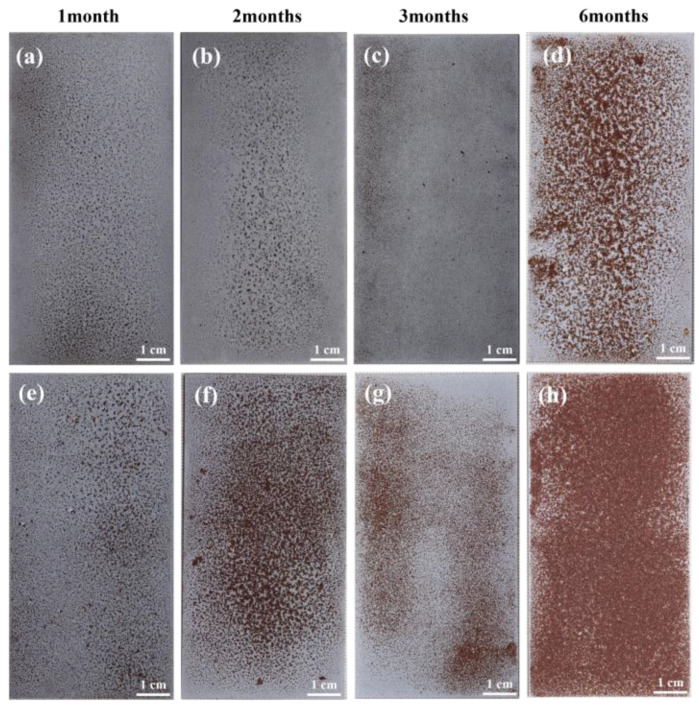
Macroscopic morphology of steel with Zn-Al coating after corrosion for different periods under varying temperature conditions. (**a**–**d**) 20 °C/75% RH; (**e**–**h**) 40 °C/75% RH.

The macroscopic morphology of flat specimens with Zn-Al coatings after exposure to different corrosion periods under conditions of 20 °C/75% RH and 40 °C/75% RH is shown in Figure 3. In the initial stage of corrosion (one month), significant pitting corrosion is observed under both temperature conditions. A large number of localized red rust spots, distributed in patches, are visible on the specimen surfaces, while areas without red rust exhibit a small amount of white corrosion products. Notably, the density of red rust coverage at 40 °C is significantly higher than that at 20 °C, indicating that the elevated temperature accelerates the localized failure process of the coating, allowing corrosive media to penetrate the coating and initiate electrochemical reactions with the substrate. As the corrosion period extends, the area of red rust on the specimen surfaces gradually increases. After six months of corrosion, the red rust coverage on the 20 °C/75% RH specimen exceeds 50% of the total surface area, while the 40 °C/75% RH specimen exhibits nearly full-surface red rust coverage. These results demonstrate that the increase in temperature significantly exacerbates the corrosion failure rate of the Zn-Al coating and the oxidation degree of the substrate metal.

### 3.4. Microscopic Morphology of Corrosion

#### 3.4.1. Surface Morphology

**Figure 4:**
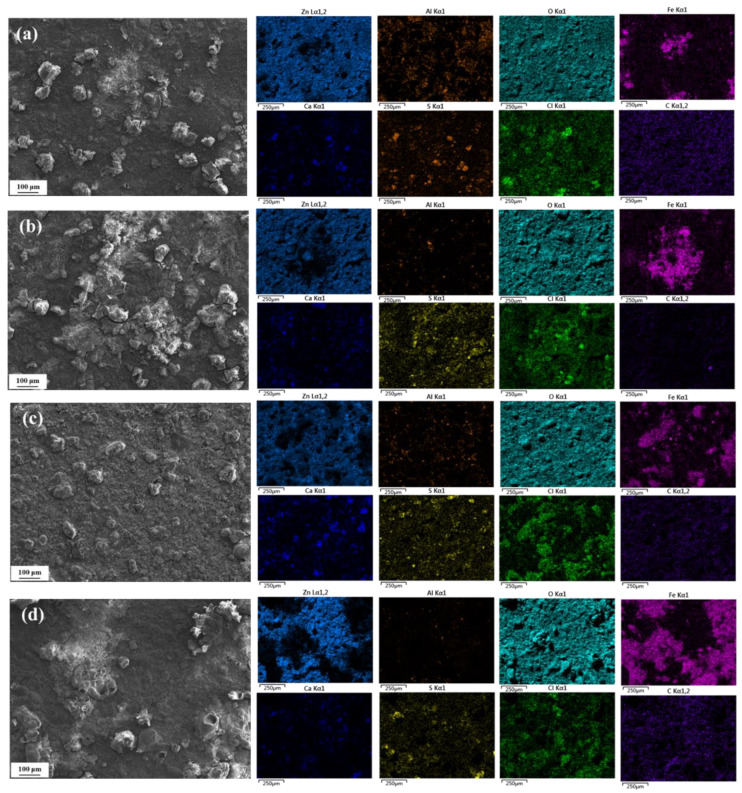
Surface morphology of material with Zn-Al coating after different corrosion periods at 20 °C/75% RH. (**a**) 1 month; (**b**) 2 months; (**c**) 3 months; (**d**) 6 months.

Figure 4 shows the surface microscopic morphology of spring steel specimens with Zn-Al coating after being corroded for 1, 2, 3, and 6 months in a constant temperature and humidity environment of 20 °C/75% RH. SEM observations reveal that after one month of corrosion, the specimen surface exhibits distinct granular substances. Combined with the results of EDS elemental mapping analysis [10], the primary chemical components are identified as S and Ca. It is speculated that these substances may have formed due to the deposition and subsequent deliquescence of salts on the specimen surface, leading to the combination of cations and anions to generate insoluble compounds, such as CaSO_4_ and CaCO_3_, which remain on the specimen surface. Simultaneously, significant enrichment of Zn, Al, and O elements is detected on the surface, with localized regions showing the presence of Fe. This indicates that after one month of corrosion, the coating in some areas is compromised, exposing the internal substrate to the corrosive medium and resulting in corrosion. The primary corrosion products on the specimen surface are identified as zinc oxides, along with minor amounts of aluminum and iron oxides.

As the corrosion period extended to six months, the surface morphology of the specimen underwent significant changes. In addition to granular deposits, a large number of typical mound-like protrusions appeared. EDS results show that the Zn and Al elemental content on the surface of the specimen decreases gradually with the extension of corrosion time; moreover, the decrease in Zn is not as obvious as that of Al because Zn preferentially forms layered structures, such as Zn_5_(OH)_8_Cl_2_ and ZnO, in the Cl^−^ environment, and the solubility of such compounds is low, and a dense passivation film can be formed on the surface, which significantly slows down the further dissolution of Zn. Al(OH)_3_ and AlOOH formed by Al in the presence of Cl^−^ are easily soluble under acidic conditions, and Cl^−^ can penetrate the oxide film and cause pitting corrosion, while the content of Fe elements progressively increases, covering nearly half of the detected area. This indicates that the integrity of the coating deteriorates over time, leading to a gradual decline in its protective effect on the internal substrate, which results in intensified substrate corrosion, manifesting as the formation of more red rust on the specimen surface. Simultaneously, a significant enrichment of Cl elements is observed on the specimen surface, particularly in regions with Fe enrichment. This suggests that Cl^−^ plays a crucial role in the corrosion process of the Zn-Al coating, preferentially attacking weak areas of the coating, initiating pitting corrosion, and accelerating the corrosion process of the substrate.

**Figure 5:**
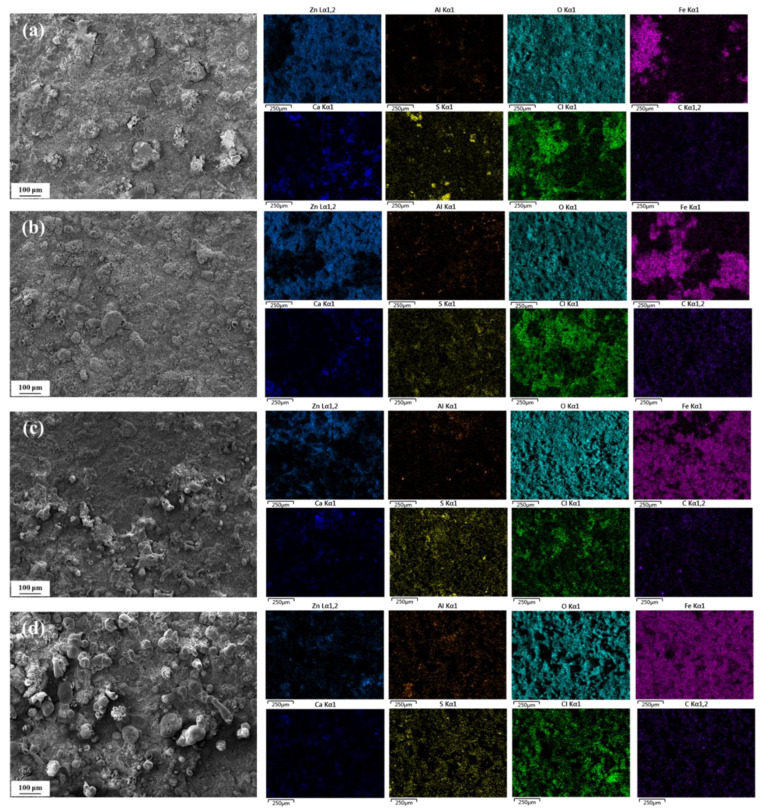
Surface morphologies of material with Zn-Al coating after different corrosion periods at 40 °C/75% RH. (**a**) 1 month; (**b**) 2 months; (**c**) 3 months; (**d**) 6 months.

Figure 5 shows the surface micro-morphology of spring steel specimens with a Zn-Al coating after being corroded for 1, 2, 3, and 6 months at 40 °C/75% RH. Similar to the conditions at 20 °C, insoluble particles are still observed on the specimen surface after one month of corrosion at 40 °C. However, the difference is that distinct mound-like protrusions appear as early as two months of corrosion at 40 °C, and their number continues to increase with prolonged corrosion time, accompanied by an increase in size. By the time the corrosion period reaches six months, almost no insoluble particles are detected on the specimen surface, and the protrusions have interconnected to form a continuous overlayer.

EDS analysis reveals that the content of Zn and Al elements in the coating exhibits a significant decreasing trend with prolonged corrosion time, while the content of Fe elements gradually increases. When the corrosion period reaches six months, the detected area is almost entirely covered by Fe elements, with Zn and Al only remaining in localized micro-regions, indicating that the coating is nearly fully consumed and has lost its protective function for the substrate. Simultaneously, a substantial accumulation of Cl elements was detected on the specimen surface, which can promote the migration of metal ions by forming local micro-galvanic cells, thereby accelerating the corrosion process of both the coating and the substrate material. Comparing the EDS results at 20 °C and 40 °C for the same corrosion periods, the remaining coating at the higher temperature exhibits lower Zn and Al content and higher Fe content, further demonstrating that elevated temperatures exacerbate the consumption of the coating and accelerate the corrosion process of the substrate.

#### 3.4.2. Cross-Sectional Morphology

To further understand the consumption of the coating and the corrosion condition of the internal substrate of the Zn-Al-coated spring steel specimens under different conditions and corrosion periods, cross-sectional morphology characterization was conducted on flat specimens embedded in resin after exposure to various temperature conditions and corrosion durations.

**Figure 6:**
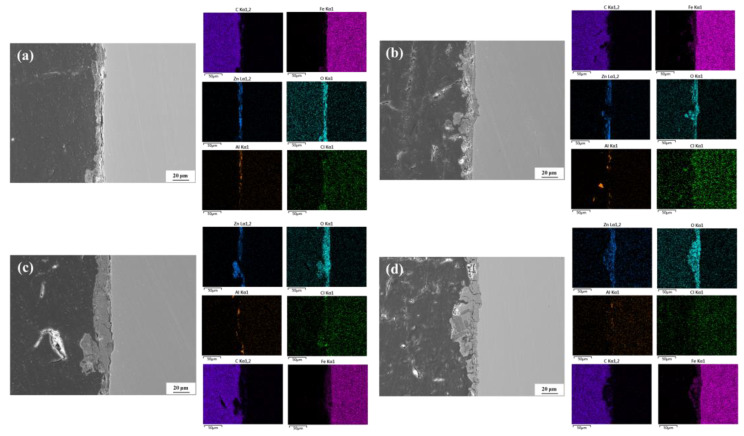
Cross-sectional morphologies of Zn-Al-coated specimens after exposure to 20 °C/75% RH conditions for different corrosion periods. (**a**) 1 month; (**b**) 2 months; (**c**) 3 months; (**d**) 6 months.

Figure 6 presents the cross-sectional morphologies and EDS elemental mapping of the Zn-Al-coated spring steel specimens after exposure to 20 °C/75% RH environmental conditions for different corrosion periods. It can be observed that, compared to the relatively intact coating before corrosion, the coatings after corrosion exhibit varying degrees of damage. As the corrosion period extends, the thickness of the corrosion product layer on the specimen surface shows an increasing trend, and the initially smooth substrate surface gradually evolves into localized pitting morphology. Combined with the EDS results, significant enrichment of O, Cl, and Fe elements was detected at the initial coating locations, while the relative contents of Zn and Al elements gradually decrease with prolonged corrosion time. The deliquescence of deposited salts forms an electrolyte solution, in which Zn and Al in the coating act as anodes and undergo preferential electrochemical corrosion. Under the combined action of aggressive ions, such as Cl^−^, and solid particles, the weak regions of the coating (e.g., the overlapping interfaces of Zn and Al) are damaged. Once the integrity of the coating is compromised, the electrolyte solution penetrates through micro-defect channels to the metal substrate interface, initiating corrosion reactions. As the corrosion process progresses, the continuous consumption of the remaining intact coating and the depth-extended damage areas jointly contribute to the non-uniform evolution of the surface layer thickness.

**Figure 7:**
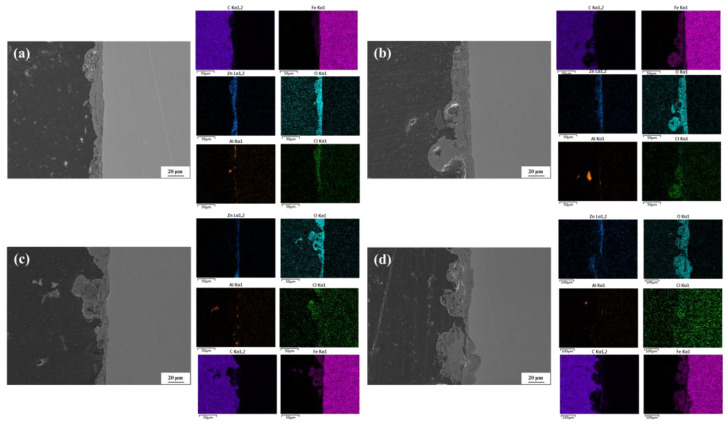
Cross-sectional morphologies of Zn-Al-coated specimens after exposure to 40 °C/75% RH conditions for different corrosion periods. (**a**) 1 month; (**b**) 2 months; (**c**) 3 months; (**d**) 6 months.

Figure 7 presents the cross-sectional morphologies and EDS elemental mapping of the Zn-Al-coated spring steel specimens after exposure to 40 °C/75% RH conditions for different corrosion periods. Under the same corrosion duration, the surface corrosion layer thickening effect is more pronounced in the 40 °C specimens, with significantly reduced contents of Zn and Al elements in the coating, indicating an accelerated consumption rate of the coating. After one month of corrosion, the surface layer thickness of the specimen is approximately 10 μm, which is notably higher compared to that at 20 °C. As the corrosion period extends to two months, the surface layer thickness increases, and localized protrusions become evident, suggesting the occurrence of significant pitting corrosion at these sites. With further prolongation of the corrosion period, the pitting phenomenon becomes more pronounced. By six months of corrosion, the depth of the corrosion pits exceeds 20 μm. These results demonstrate that the increase in temperature enhances the electrochemical activity of Zn and Al, significantly accelerating the degradation process of the coating. This leads to a continuous decline in the structural integrity of the coating over time, gradually weakening its protective effect on the substrate.

### 3.5. Corrosion Product Analysis

#### 3.5.1. XRD Analysis

To investigate the composition of corrosion products on specimens with Zn-Al coatings after corrosion, block samples of approximately 1 cm^2^ were extracted from flat panels under various conditions and corrosion periods for XRD testing.

**Figure 8:**
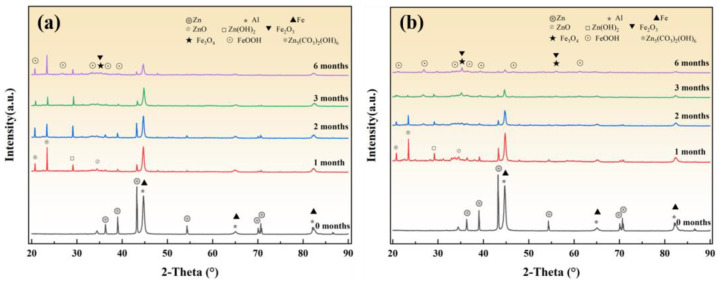
XRD diffraction patterns of material with Zn-Al coating after corrosion under different temperature conditions. (**a**) 20 °C/75% RH; (**b**) 40 °C/75% RH.

Figure 8 presents the XRD diffraction patterns of spring steel material coated with Zn-Al after exposure to corrosion at 20 °C/75% RH and 40 °C/75% RH for varying periods. The result distinctly reveals that the primary phases of the corrosion products consist of ZnO, Zn(OH)_2_, Zn_5_(CO_3_)_2_(OH)_6_, Fe_3_O_4_, Fe_2_O_3_, and FeOOH. Due to the low aluminum content in the coating, its corrosion products were not detected in the XRD spectra [17]. Compared to the original coating, after one month of corrosion at 20 °C, the intensity of the characteristic diffraction peaks of Zn and Al significantly decreases, accompanied by the formation of ZnO, Zn(OH)_2_, and Zn_5_(CO_3_)_2_(OH)_6_. Simultaneously, the characteristic peaks of iron oxides and hydroxides emerge, indicating that the coating has partially deteriorated under the combined effects of aggressive ions such as Cl^−^ and SO_4_^2−^, as well as solid particles deposited on the sample surface. As the corrosion period extends to six months, the content of Zn and Al gradually decreases, but the corrosion products of Zn are still detectable, suggesting that the Zn-Al coating has not been entirely consumed and still provides some level of protection to the substrate. However, after six months of corrosion at 40 °C, the characteristic peaks of Zn corrosion products have nearly disappeared, and the intensity of Fe characteristic peaks significantly increases, indicating accelerated consumption of the coating and severe corrosion of the steel substrate.

#### 3.5.2. Raman Analysis

**Figure 9:**
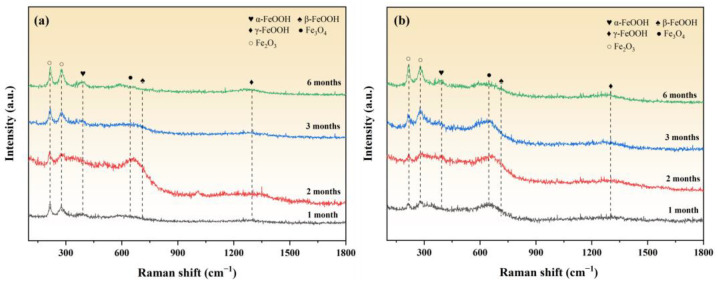
Raman spectra of the red rust areas on the material with a Zn-Al coating after corrosion under different temperature conditions. (**a**) 20 °C/75% RH; (**b**) 40 °C/75% RH.

Based on the results of the surface macroscopic morphology, red rust appeared on the surface of the specimen after corrosion. To investigate the composition of the red rust, Raman tests were conducted on the red rust areas of samples corroded under different conditions. Figure 9 shows the Raman spectra of the corrosion products at the red rust sites on the material with a Zn-Al coating after corrosion.

The main components of the red rust products under both temperature conditions are α-FeOOH, β-FeOOH, γ-FeOOH, Fe_3_O_4_, and Fe_2_O_3_, which are consistent with the corrosion products of the bare metal specimen [18]. As the corrosion time extends, the characteristic diffraction peak intensities of Fe_2_O_3_ and α-FeOOH gradually increase, reaching their highest levels at six months of corrosion [19]. This indicates that the metastable corrosion products gradually transform into thermodynamically stable phases over time. Meanwhile, the characteristic peak intensity of Fe_3_O_4_ under the 40 °C corrosion condition is significantly higher than that at 20 °C. This is primarily due to the elevated temperature accelerating the corrosion kinetics, prompting the rapid deposition of corrosion products to form a dense layer on the specimen surface. This dense layer inhibits the diffusion of environmental oxygen into the substrate, thereby promoting the formation of Fe_3_O_4_ under oxygen-deficient conditions [20,21].

### 3.6. Electrochemical Testing

**Figure 10:**
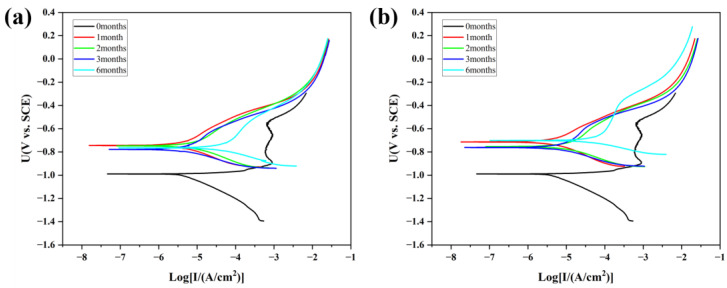
Polarization curves of specimens with Zn-Al coating after corrosion under different temperature conditions. (**a**) 20 °C/75% RH; (**b**) 40 °C/75% RH.

Figure 10 shows the polarization curves of the spring steel specimens with a Zn-Al coating under 20 °C/75% RH and 40 °C/75% RH conditions after different corrosion cycles. The self-corrosion potential and self-corrosion current density, obtained by Tafel extrapolation fitting, are presented in Table 1.

From the fitting results of the polarization curves, it can be observed that the self-corrosion potential of the specimens after corrosion under different conditions is higher compared to the uncorroded specimens. This phenomenon can be attributed to two main factors. Firstly, metals such as Zn and Al in the Zn-Al coating have relatively low potentials and are prone to preferential corrosion [22]. This leads to the formation of corrosion products, such as zinc carbonate hydroxide and zinc oxide, which have higher and more stable potentials than pure Zn and Al, adhering to the surface of the specimens. Secondly, aggressive media penetrate the coating through weak points (such as the interfaces between zinc and aluminum particles), causing localized corrosion of the substrate. This results in the formation of localized rust spots, and since the potential of iron rust is higher than that of the 60Si2MnA substrate, the overall self-corrosion potential is elevated.

Three months prior to corrosion, the self-corrosion current density exhibits a slight decrease compared to its initial state, indicating that although pitting has occurred within the internal matrix during the early stages of corrosion, the combined effect of the remaining coating and corrosion products still provided a commendable protective barrier to the internal matrix [23]. However, after six months of corrosion, under the influence of aggressive ions such as Cl^−^ and SO_4_^2−^, more of the coating is compromised, leading to the gradual corrosion of the steel substrate and a significant decline in the protective efficacy of the coating. The self-corrosion current density increases nearly tenfold compared to after three months of corrosion. The self-corrosion current of the specimens after six months of corrosion at 40 °C is slightly higher than at 20 °C, which is consistent with the macroscopic observation that more red rust spots and more severe coating damage are produced at 40 °C.

**Figure 11:**
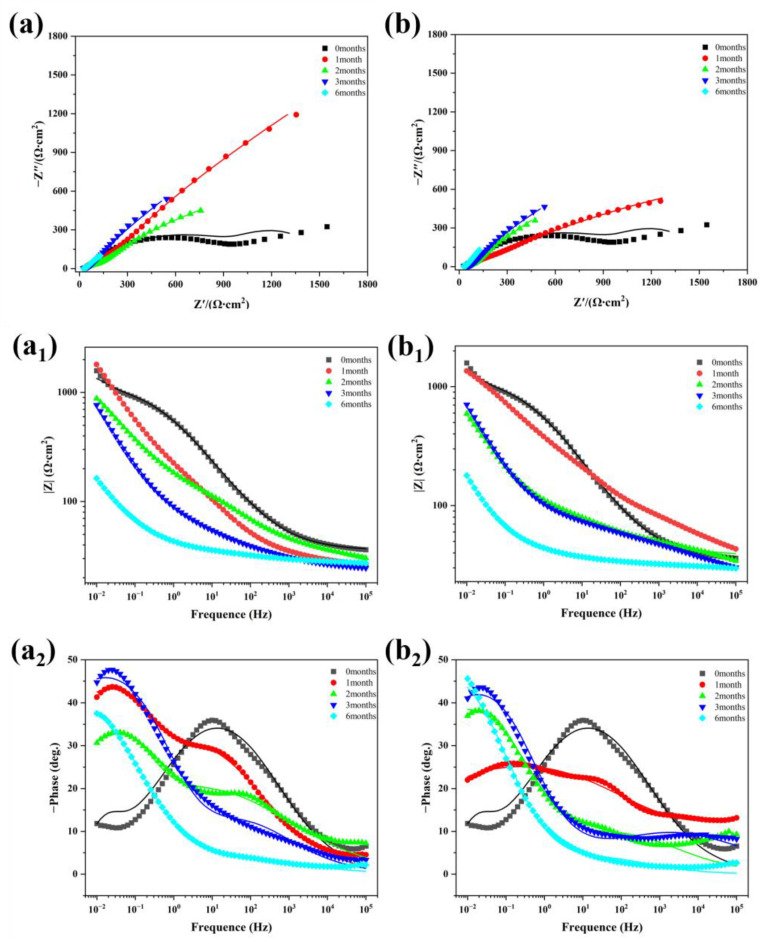
Impedance spectra of Zn-Al-coated specimens at different periods of corrosion in simulated atmospheric environment. (**a**–**a_2_**) 20 °C/75% RH; (**b**–**b_2_**) 40 °C/75% RH.

**Figure 12:**
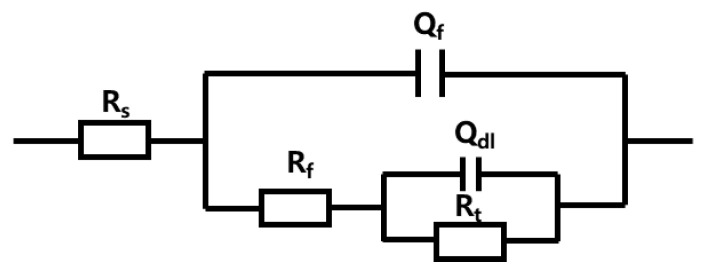
Equivalent circuit diagram for EIS fitting.

Figure 11 presents the Nyquist and Bode plots of spring steel specimens with a Zn-Al coating before and after corrosion, where the scatter points represent the experimental test data and the line graphs depict the fitted data based on the equivalent circuit. Figure 12 illustrates the equivalent circuit diagram used for the electrochemical impedance fitting of the Zn-Al-coated specimens, with the model of the equivalent circuit being R_s_(Q_f_(R_f_(Q_dl_R_ct_))). In this model, R_s_ denotes the solution resistance, R_f_ represents the corrosion product film resistance, the phase element Q_f_ corresponds to the corrosion product film capacitance, R_ct_ stands for the charge transfer resistance at the interface between the electrolyte solution and the substrate, and Q_dl_, as a phase element, signifies the double-layer capacitance on the metal surface [24].

Table 2 presents the fitted data from the electrochemical impedance spectra of Zn-Al-coated specimens after different periods of corrosion under varying temperature conditions. Compared to the uncorroded specimens, the resistance of the zinc–aluminum coating in the corroded specimens gradually decreases. Additionally, for the same corrosion period, the resistance of specimens corroded at 40 °C is lower than that of those corroded at 20 °C. These results indicate that the integrity of the coating deteriorates progressively with corrosion time, and an increase in temperature accelerates the degradation of the coating.

To evaluate the changes in corrosion resistance of specimens under different temperatures and corrosion periods, *R_p_* was used to represent the polarization resistance of the corroded specimens in the electrolyte solution system:

(1) $$R_{p}=R_{ct}+R_{f}$$

The calculation results are presented in Table 3. The polarization resistance of specimens corroded for 1–3 months increases compared to that of the uncorroded specimens, while it significantly decreases after six months of corrosion. This indicates that the corrosion resistance of the material improved during the first three months of corrosion due to the combined effect of the corrosion product film and the remaining coating. However, after six months of corrosion, the protective effect of the coating and corrosion product layer on the substrate is significantly weakened under the influence of aggressive ions, such as Cl^−^ and SO_4_^2−^, as well as solid particles, leading to a notable decline in the corrosion resistance of the material. This observation is consistent with the results obtained from the polarization curves.

The weight gain of the samples at 20 °C increased from 5.93 g/m^2^ after one month to 21.20 g/m^2^ after six months, with the thickness of the corrosion product layer increasing over time and the substrate evolving from a smooth surface to a pitted morphology. After six months of corrosion, there was an increase in the number of hill-like structures, a decrease in Zn/Al content, failure of the coating’s protective function, and an intensification of substrate pitting. For samples at 40 °C, after six months of corrosion, the weight gain reached 35.35 g/m^2^, which is 66.7% higher than at 20 °C, indicating that elevated temperatures significantly accelerate the corrosion process. The hill-like structure appeared within two months, forming a continuous coverage layer after six months, with Zn/Al nearly depleted. In the first three months of corrosion, due to the synergistic protective effect of the corrosion product film and remaining coating, the corrosion current slightly decreased; however, after six months, there was a tenfold increase in the current (40 °C > 20 °C). The *R_p_* value during the corrosion period of one to three months increased compared to the non-corroded state but dropped sharply after six months. This indicates that during the later stages of corrosion, under the influence of aggressive ions, such as Cl^−^ and SO_4_^2−^, the protective effect of the coating and the corrosion product layer on the substrate was significantly weakened, leading to a marked decline in the material’s corrosion resistance.

### 3.7. Analysis of Corrosion Mechanism

When the deposited salt undergoes deliquescence on the surface of the specimen coated with Zn-Al, a thin liquid film is formed. The Zn and Al in the coating, having lower self-corrosion potentials, act as sacrificial anodes, providing cathodic protection and being preferentially corroded. The electrochemical corrosion reactions related to Zn are as follows:

(2) $$Anode\;:Zn\to {Zn}^{2+}+2e^{-}$$

(3) $$Cathode\;:O_{2}+2H_{2}O+4e^{-}\to 4{OH}^{-}$$

(4) $$Overall\;reaction\;:2Zn+O_{2}+2H_{2}O\to 2Zn{\left(OH\right)}_{2}\to ZnO\cdot H_{2}O$$

Meanwhile, in a humid environment, *Zn* reacts with *O*_2_ and *CO*_2_ in the air to form a relatively stable white corrosion product–basic zinc carbonate, which provides a good protective effect on the substrate:

(5) $$5Zn+O_{2}+2{CO}_{2}+4H_{2}O\to {Zn}_{5}{\left({CO}_{3}\right)}_{2}{\left(OH\right)}_{6}$$

However, since the electrolyte solution also contains *Cl*^−^ or *SO*_4_^2−^ ions, *Zn*^2+^ may also combine with them to form soluble ZnCl_2_ or ZnSO_4_. Such corrosion products do not provide good protection to the substrate [25].

Moreover, the Al present in the coating also undergoes corrosion, forming Al(OH)_3_ or Al_2_O_3_. Due to the relatively low content of Al in the coating, the resulting corrosion products were not detected by XRD. In general, in atmospheric conditions, the oxides of Al exhibit high passivity and provide better protection; even a nanometer-thick layer of oxide or hydroxide can offer effective protection. However, the presence of Cl^−^ and SO_4_^2−^ ions can disrupt the passive film on the Al surface, promoting further corrosion reactions [26,27].

**Figure 13:**
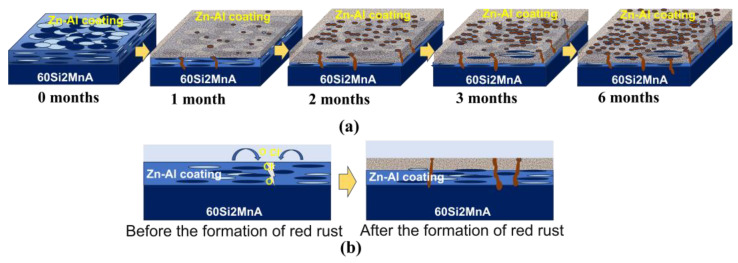
Schematic diagram of corrosion evolution process of spring steel samples with Zn-Al coatings. (**a**) Three-dimensional schematic; (**b**) Two-dimensional schematic.

The evolution of the corrosion process at different temperatures is basically the same, and temperature was used as an equivalent acceleration factor to improve the efficiency of the experiment. The specific evolution is as follows (Figure 13):

Based on the analysis of the microscopic morphology and corrosion products, the surface of the coating before corrosion primarily consists of overlapping Zn-rich and Al-rich phases distributed in a lamellar structure, with diameters ranging from several micrometers to tens of micrometers and thicknesses of less than 1 micrometer. In the initial stage of corrosion, due to the lower potential and higher reactivity of Zn, it preferentially corrodes to form Zn(OH)_2_ or ZnO, which subsequently reacts with CO_2_ in the air to produce relatively protective zinc carbonate hydroxide, manifesting as white corrosion products. However, due to the presence of harmful ions, such as Cl^−^ and SO_4_^2−^, in the deposited salts, the zinc carbonate hydroxide formed on the sample surface is not dense [28] and is intermixed with soluble and less protective corrosion products, like ZnCl_2_ or ZnSO_4_. As the corrosion process continues, harmful ions, such as Cl^−^ and O, can continuously penetrate the interior of the coating through these areas or weak points in the coating. With prolonged corrosion time, the thickness of the surface layer (including the remaining coating and insoluble corrosion products) gradually increases, and the weight change of the sample also indicates a weight gain after corrosion. When the corrosive substances penetrate the coating and reach the substrate, they cause substrate corrosion, forming iron oxyhydroxides, hydroxides, and oxides. Red rust can be observed emerging from the coating at these locations in the surface morphology [29].

## 4. Conclusions

(1) The weight gain of spring steel samples with Zn-Al coatings increases with prolonged corrosion time under both 20 °C/75% RH and 40 °C/75% RH conditions, with the weight gain rate being higher at 40 °C than at 20 °C. Combined with the macroscopic morphology, the consumption of the Zn-Al coating gradually increases over time, and its protective effect on the internal substrate weakens. At 40 °C, the coating essentially loses its protective effectiveness after six months of corrosion. This indicates that temperature can serve as an equivalent acceleration factor to enhance the efficiency of testing for samples with Zn-Al coatings.
(2) According to the XRD and Raman results, the main phases of the corrosion products include ZnO, Zn(OH)_2_, Zn_5_(CO_3_)_2_(OH)_6_, Fe_3_O_4_, Fe_2_O_3_, FeOOH, etc., and the presence of Al corrosion products cannot be ruled out. As the corrosion time progresses, the characteristic peaks of Zn corrosion products gradually weaken, while those of Fe corrosion products gradually strengthen.
(3) Based on the electrochemical test results, the corrosion potential of samples after corrosion under different conditions increases compared to uncorroded samples, indicating reduced corrosion sensitivity. In the first three months of corrosion, even though obvious pitting occurs on the sample surface, the self-corrosion current density slightly decreases compared to before corrosion due to the protective effect of the remaining coating and corrosion products, leading to improved corrosion resistance. After six months of corrosion and under the combined influence of Cl^−^ and solid particles deposited on the surface, the integrity of the coating and the substrate corrosion product layer deteriorates, and the self-corrosion current density increases nearly tenfold, resulting in a significant decline in corrosion resistance.

## Figures and Tables

**Table 1:** Fitted parameters of potentiodynamic polarization curves for specimens with Zn-Al coating.

Specimen Type	E_corr_ (V)	I_corr_ (A·cm^−2^)
Uncorroded	−1.029	7.23 × 10^−6^
20 °C/75% RH 1 month	−0.745	3.42 × 10^−6^
20 °C/75% RH 2 months	−0.740	6.10 × 10^−6^
20 °C/75% RH 3 months	−0.766	4.66 × 10^−6^
20 °C/75% RH 6 months	−0.768	3.49 × 10^−5^
40 °C/75% RH 1 month	−0.713	3.81 × 10^−6^
40 °C/75% RH 2 months	−0.754	7.73 × 10^−6^
40 °C/75% RH 3 months	−0.760	4.45 × 10^−6^
40 °C/75% RH 6 months	−0.704	4.24 × 10^−5^

**Table 2:** Fitted data of impedance spectra for Zn-Al-coated specimens in simulated environment.

Sample	R_s_/ (Ω·cm^2^)	R_f_/ (Ω·cm^2^)	Q_f_		R_t_/ (Ω·cm^2^)	Qdl	
			Y_0_/(Ω^−1^·cm^2^·s^n^)	n_f_		Y_0_/(Ω^−1^·cm^2^·s^n^)	n_dl_
Uncorroded	35.58	1195.00	4.86 × 10^−4^	0.52	1805.30	2.54 × 10^−2^	0.68
20 °C 1 month	26.06	916.90	0.71 × 10^−3^	0.46	9097.90	8.27 × 10^−4^	0.83
20 °C 2 months	28.01	489.30	2.40 × 10^−3^	0.34	3299.13	1.55 × 10^−3^	0.79
20 °C 3 months	24.33	47.34	2.00 × 10^−3^	0.47	7475.49	4.85 × 10^−3^	0.64
20 °C 6 months	26.99	11.43	6.74 × 10^−3^	0.42	873.27	2.44 × 10^−2^	0.54
40 °C 1 month	27.29	629.17	1.67 × 10^−4^	0.58	8136.17	1.52 × 10^−3^	0.33
40 °C 2 months	37.74	258.03	1.67 × 10^−3^	0.42	1928.07	6.09 × 10^−3^	0.57
40 °C 3 months	22.95	61.28	1.29 × 10^−3^	0.34	4438.70	5.87 × 10^−3^	0.64
40 °C 6 months	30.42	10.86	6.84 × 10^−3^	0.50	481.64	2.72 × 10^−2^	0.63

**Table 3:** *R_p_* values based on EIS fitting.

Specimen Type	*R_p_*/(Ω·cm^2^)	Specimen Type	*R_p_*/(Ω·cm^2^)
Uncorroded	3000.30	Uncorroded	3000.30
20 °C/75% RH 1 month	10,014.80	40 °C/75% RH 1 month	8765.34
20 °C/75% RH 2 months	3788.43	40 °C/75% RH 2 months	2186.10
20 °C/75% RH 3 months	7522.83	40 °C/75% RH 3 months	4499.98
20 °C/75% RH 6 months	884.70	40 °C/75% RH 6 months	492.50

## Data Availability

The original contributions presented in the study are included in the article, further inquiries can be directed to the corresponding author.
